# Academic Emergency Medicine Physicians’ Knowledge of Mechanical Ventilation

**DOI:** 10.5811/westjem.2016.2.29517

**Published:** 2016-04-26

**Authors:** Susan R. Wilcox, Tania D. Strout, Jeffrey I. Schneider, Patricia M. Mitchell, Jessica Smith, Lucienne Lutfy-Clayton, Evie G. Marcolini, Ani Aydin, Todd A. Seigel, Jeremy B. Richards

**Affiliations:** * Medical University of South Carolina, Divisions of Emergency Medicine and Pulmonary, Critical Care and Sleep Medicine, Charleston, South Carolina; † Maine Medical Center, Department of Emergency Medicine, Portland, Maine; ‡ Boston Medical Center, Department of Emergency Medicine, Boston, Massachusetts; § Alpert Medical School of Brown University, Department of Emergency Medicine, Providence, Rhode Island; ¶ Baystate Medical Center, Department of Emergency Medicine, Springfield, Massachusetts; || Yale University School of Medicine, Departments of Emergency Medicine and Neurology, Divisions of Neurocritical Care and Emergency Neurology and Surgical Critical Care, New Haven, Connecticut; # Yale University School of Medicine, Department of Emergency Medicine, New Haven, Connecticut; ** Department of Emergency Medicine and Critical Care, Kaiser Permanente East Bay, Oakland and Richmond Medical Centers, California; †† Medical University of South Carolina, Division of Pulmonary, Critical Care and Sleep Medicine, Charleston, South Carolina

## Abstract

**Introduction:**

Although emergency physicians frequently intubate patients, management of mechanical ventilation has not been emphasized in emergency medicine (EM) education or clinical practice. The objective of this study was to quantify EM attendings’ education, experience, and knowledge regarding mechanical ventilation in the emergency department.

**Methods:**

We developed a survey of academic EM attendings’ educational experiences with ventilators and a knowledge assessment tool with nine clinical questions. EM attendings at key teaching hospitals for seven EM residency training programs in the northeastern United States were invited to participate in this survey study. We performed correlation and regression analyses to evaluate the relationship between attendings’ scores on the assessment instrument and their training, education, and comfort with ventilation.

**Results:**

Of 394 EM attendings surveyed, 211 responded (53.6%). Of respondents, 74.5% reported receiving three or fewer hours of ventilation-related education from EM sources over the past year and 98 (46%) reported receiving between 0–1 hour of education. The overall correct response rate for the assessment tool was 73.4%, with a standard deviation of 19.9. The factors associated with a higher score were completion of an EM residency, prior emphasis on mechanical ventilation during one’s own residency, working in a setting where an emergency physician bears primary responsibility for ventilator management, and level of comfort with managing ventilated patients. Physicians’ comfort was associated with the frequency of ventilator changes and EM management of ventilation, as well as hours of education.

**Conclusion:**

EM attendings report caring for mechanically ventilated patients frequently, but most receive fewer than three educational hours a year on mechanical ventilation, and nearly half receive 0–1 hour. Physicians’ performance on an assessment tool for mechanical ventilation is most strongly correlated with their self-reported comfort with mechanical ventilation.

## INTRODUCTION

Although emergency physicians frequently intubate critically ill patients in the emergency department (ED), management of mechanical ventilation has traditionally not been emphasized in emergency medicine (EM) practice.^1^–^4^ In a previous study of EM residents, we found that while EM residents report caring for mechanically ventilated patients frequently in the ED, they received few hours of education on mechanical ventilation.^5^ We measured residents’ knowledge of mechanical ventilation and found that their performance on our assessment tool was only correlated with their self-reported comfort with caring for mechanically ventilated patients.^5^

Management of positive-pressure ventilation can influence outcomes of critically ill patients for several conditions commonly encountered in EM practice.^6^–^11^ Patients with asthma are at high risk of complications and deterioration once intubated.^8^ Low-tidal volume ventilation improves mortality in patients with acute respiratory distress syndrome (ARDS).^11^ Careful management of oxygenation and ventilation by emergency care providers improves outcomes in intubated patients with traumatic brain injury.^12^,^13^ Furthermore, with increasing ED length of stays, emergency physicians may be responsible for management of mechanically ventilated patients for prolonged periods.^14^–^16^ Even for patients who are in the ED only briefly, ventilator-induced lung injury can occur in as little as 20 minutes.^17^

We designed this study to quantify academic EM attendings’ experience and knowledge regarding mechanical ventilation. We surveyed EM attendings to assess how frequently they receive education on mechanical ventilation, the frequency with which they care for mechanically ventilated patients in the ED, and their subjective comfort with managing mechanically ventilated patients. In addition, we used a knowledge assessment tool to characterize attendings’ knowledge regarding mechanical ventilation involving common emergency scenarios. We hypothesized that attendings with the most experience in managing mechanical ventilators in the ED would achieve higher scores on the assessment tool.

## METHODS

### Survey instrument development

Previously, to quantify EM residents’ training experiences, we developed a five-point Likert scale survey tool to assess residents’ hours of education on mechanical ventilation, frequency with which they care for mechanically ventilated patients, and their comfort with managing ventilators. Details of survey development have been previously described.^5^ We modified the EM residents’ survey to better reflect attendings’ practice. Survey responses were dichotomized as affirmative or negative: responses “often” and “frequently” were defined as affirmative, while “never,” “rarely,” or “don’t know” were defined as negative. Any responses left blank were scored as “don’t know.”

### Assessment instrument development

A project team with backgrounds in EM, critical care, and educational survey development^18^,^19^ generated an assessment instrument with questions specific to EM. We created a series of questions involving key principles consistent with outlined objectives for resident education in mechanical ventilation,^20^ and the content was modified to be relevant to management of mechanically ventilated patients in the ED. Clinical scenarios emphasized emergency management of ventilated patients with asthma, ARDS, and traumatic brain injury, as evidence supports the importance of conscientious ventilator management for these conditions. 6,8,11,12,21–28 Questions were iteratively reviewed and edited by subject experts to optimize content, length, and relevance to the assessment tool’s goals, as previously described.^5^

### Study Protocol

Finalized versions of the survey and assessment tool were administered anonymously using REDCap (Nashville, TN) electronic data capture tools.^29^ REDCap (Research Electronic Data Capture) is a secure, web-based application designed to support data capture for research studies, providing an interface for validated data entry.

The survey and assessment tool were distributed by email to all EM attendings affiliated with the key teaching hospitals for seven EM residency training programs in the northeastern United States. The survey was sent via email invitation to attendings by a local site investigator once a week for three weeks in March and April 2015. The study protocol was approved by the institutional review boards of all participating institutions. Consent was obtained from participants at the time of participation, as the survey introduction stated that partaking of the survey indicated consent.

### Data Analysis

Study data were exported into Microsoft Excel (Microsoft Corp., Redmond, WA) and then transferred into SPSS (v. 11.0, SPSS, Inc., Chicago, IL) for analysis. For all variables, we excluded missing data on a case-by-case basis.

For the purposes of this study, we assumed the correct response rate for the assessment tool (test score) to be a surrogate for knowledge of mechanical ventilation. We examined the continuous outcome variable test score for normality in two ways. First, the outcome was examined visually using histograms and normal quantile-quantile plots. Then, Pearson’s second skewness coefficient was computed, revealing mild skew to the left, Sk_2_=−0.68. Survey data regarding study participants and characteristics of their training programs, mechanical ventilation educational experiences, and ventilator management experience were summarized using descriptive statistics. We used one-way analysis of variance to assess for differences in total test score across participating institutions. Tukey’s honest significant difference (HSD) was employed to assess for differences between institutional pairs.

As our hypothesis was that attendings with the most exposure to managing mechanical ventilators in the ED would perform better on the knowledge assessment tool, we examined the relationship between these variables in several ways. Ordinary least squares regression analyses were performed with total test score serving as the outcome variable. The frequency with which attendings managed mechanically ventilated patients was the predictor variable. To examine the relationship between these variables after controlling for other variables significantly correlated to test score in simple correlation analysis (Spearman’s ρ), we employed hierarchical multiple regression models using the additional predictors program affiliation, completion of an EM residency training program, residency program emphasis on mechanical ventilator management, working in a setting where emergency physicians bear primary responsibility for ventilator management, and subjective comfort with managing mechanically ventilated ED patients. Exploratory regression analyses were then conducted to determine which variables, alone and in combination, were the strongest predictors of total test score.

In addition to assessing normality, we evaluated additional linear regression assumptions using residual analyses and assessment of influence diagnostics. Multicollinearity was evaluated using variance inflation factors, which were all well below recommended cut points. We performed multivariate logistic regression analyses to evaluate the extent to which completion of an EM residency, program affiliation, residency program emphasis on mechanical ventilation management, working in a setting where an emergency physician bears primary responsibility for ventilator management, and level of comfort with managing ventilated patients influenced attendings’ self-reported comfort with managing mechanically ventilated patients. Coefficient estimates, adjusted odds ratios (aORs) and 95% confidence intervals (CIs) are reported for each variable. We accepted an alpha of less than 0.05 as statistically significant.

## RESULTS

### Characteristics of the Study Subjects

Study surveys were distributed to 394 academic EM attendings, with 211 responding (response rate=53.6%). One physician completed the survey questions without answering any questions from the knowledge assessment tool, and seven other physicians did not fully complete the knowledge assessment tool. The response rate from the institutions ranged from 23.4 to 91.3%. The number of years as an attending emergency physician was well-distributed among the respondents, from 0–2 up to >15 years (Table 1).

### Educational Opportunities and Experience Managing Ventilated Patients

Overall, study participants reported few educational opportunities regarding mechanical ventilation, as 158 attendings (74.5%) reported receiving three or fewer hours of ventilation-related education from EM sources over the past year and 98 (46%) reported receiving between 0–1 hour of education. Responses regarding educational experiences varied significantly among respondents from individual institutions (χ^2^=36.761, df =24, p=0.046). Similarly, only 29 (15%) respondents of those who completed an EM residency recalled mechanical ventilation being often or frequently emphasized in their training.

Conversely, attendings reported frequently caring for mechanically ventilated patients in the ED. Sixty-four percent (n=136) reported that they care for four or more ventilated patients per month, and 18.5% (n=39) reported caring for 10 or more. Furthermore, 56% of respondents stated that mechanically ventilated patients rarely have changes made to their ventilator while they are in the ED. Sixty percent (n=126) of participants described feeling comfortable caring for mechanically ventilated ED patients “often” or “frequently”; whereas 38.2% (n=81) described “never” or “rarely” feeling comfortable managing these patients. Only 27.9% (n=59) described management of the ventilator as the responsibility of an emergency physician (resident or attending) at their institution, while 69% (n=145) identified the respiratory therapist (RT) as being primarily in charge of ventilator management (Table 1).

### Ventilator Management Knowledge

The overall correct response rate for the nine-question assessment tool was 73.4%, standard deviation (SD)±19.9%. Of the 210 attendings who completed at least part of the assessment tool, 124 (59%) achieved a score of at least 70%. Significant differences in total test scores were noted between institutions (F=4.592, p<0.001). Post-hoc analysis revealed statistically significant differences in total test score between participants from the institution with the lowest mean score and those from three other institutions (p<0.001, p=0.015, and p=0.039). The relationship between participants’ years as an attending physician and scores on the knowledge assessment was not significant. Correlation analysis revealed statistically significant relationships between total test score and completion of an EM residency, program affiliation, residency program emphasis on mechanical ventilation management, working in a setting where an emergency physician bears primary responsibility for ventilator management, and level of comfort with managing ventilated patients. Relationships between total test score and having completed training in a non-EM residency, having completed a fellowship, hours of mechanical ventilation education, the frequency of managing ventilated patients, the frequency of ED-based ventilator changes were not significant (Table 2).

### Multivariate Results

After adjusting for the effects of completion of an EM residency, program affiliation, residency program emphasis on mechanical ventilation management, working in a setting where an emergency physician bears primary responsibility for ventilator management, and level of comfort with managing ventilated patients, multivariable regression modelling determined that self-reported frequency of managing mechanically ventilated patients was not a significant predictor of total test score (t=0.163, p=0.871). Exploratory regression analyses revealed that the strongest and only significant predictor of total test score was attendings’ self-reported confidence in caring for mechanically ventilated patients (F=22.266, p<0.001). On average, test scores increased by approximately eight points (95% CI [4.5–10.9] points, p=0.001) when attendings reported feeling comfortable managing ventilated patients “often” or “frequently.” The addition of any other predictor variables, alone or in combination, did not produce a more parsimonious model.

Exploratory logistic regression modeling revealed that four variables were statistically significantly associated with attending physician comfort managing mechanically ventilated patients. Having completed an additional residency training program was most strongly associated with confidence, with an adjusted odds ratio (aOR) of 3.671 (p=0.013) for those completing an additional program as compared to those who did not. Next, working in a facility where an emergency physician bears primary responsibility for managing ventilator settings was associated with comfort, as attending physicians reporting having this role were more likely to report comfort than those who did not (aOR, 3.271, p=0.002). Working in a setting where the residency program “often” or “frequently” emphasizes ventilator management, as compared to settings that have less focus on the topic, was associated with an increased likelihood of reporting comfort (aOR, 1.732, p=0.002). Finally, attending physicians reporting four or more hours of curriculum-based ventilator education were more likely to report comfort than those reporting two or fewer hours (aOR 1.468, p=0.013). Other variables noted to be significantly correlated with physician comfort in simple correlation analysis (Table 3) did not produce significant improvements in the predictive power of the final model.

## DISCUSSION

Emergency physicians increasingly care for critically ill, mechanically ventilated patients in the ED,^30^ and due to crowding the ED length of stay is increasing.^15^ Ventilator management decisions can directly affect patient outcomes, especially in asthma, ARDS, and traumatic brain injury,^14^–^16^ conditions commonly encountered in the ED. Although mechanical ventilation has been considered integral to EM practice by key EM organizations, including the American Board of Emergency Medicine, the hours required for training and required level of proficiency are not specified.^31^ Our group has previously demonstrated that EM residents receive few hours of education on mechanical ventilation, yet report frequently caring for ventilated patients.^5^ Their self-reported comfort with caring for ventilated patients correlated with their score on the knowledge assessment instrument, and their post-graduate year, hours of residency education on mechanical ventilation, and frequency of caring for mechanically ventilated patients were associated with their comfort. To further evaluate EM physicians’ knowledge of mechanical ventilation, we subsequently assessed the attendings associated with the same residency programs previously queried.

The knowledge assessment tool used in this study was designed to reflect educational objectives for management of mechanically ventilated patients, and tested knowledge in clinical scenarios commonly encountered by physicians in the ED.^20^,^32^ The instrument was rigorously designed, pre-tested, and pilot tested to optimize psychometric and performance characteristics, and is similar to a previously validated test.^32^

In this study, academic EM attendings report that curricular time dedicated to mechanical ventilation varied among their own EM residency training programs, but very few reported frequent emphasis on the topic. Additionally, current educational opportunities on ventilation are relatively minimal, as 75% of attendings responded that they had received three or fewer hours of education on mechanical ventilation in their in the past year.

Although attendings reported few hours of education, 64% responded that they often or frequently care for intubated patients in the ED. Interestingly, while ventilated patients are common, 56% of respondents stated that patients rarely have any changes made to ventilator settings while in the ED. This may be factual, or may be a perception, as most attendings (69%) identified RTs as being primarily responsible for managing ventilators in the ED, and a minority of the attendings stated that management of the ventilator was the responsibility of an emergency physician.

Overall, the attendings performed moderately well on the knowledge assessment, with a mean score of 73.4%±19. Notably, this is identical to the prior residents’ score of 73.3%±22.^5^ In the univariate analysis, emphasis on ventilation in the physicians’ EM residency correlated with test score. Additionally, level of comfort with managing mechanically ventilated patients, completion of an EM residency, and program affiliation also correlated with the overall score. EM management of the ventilator, as opposed to RTs, was not statistically significant (p=0.053).

Multivariate analysis assessing for factors correlating with comfort in management of ventilators found that emphasis on mechanical ventilation in the attendings’ EM training, recent hours of mechanical ventilation education, frequency of ventilator changes in the ED, and EM management of mechanical ventilator all correlated with confidence in management of mechanically ventilated patients. However, frequency of managing ventilated patients did not reach statistical significance. These findings demonstrate that comfort with caring for ventilated patients may be an active process. Simply caring for ventilated patients in the ED without being a participant in decisions regarding ventilator management may not increase comfort, but EM management of the ventilator and increasing the frequency of ventilator changes in the ED does increase comfort.

Prior work regarding mechanical ventilation in the ED is limited.^33^–^35^ Despite numerous studies showing improved outcomes with low tidal volume ventilation, prior surveys of ventilation in the ED have found that only 27.1%^33^ and 55.7%^34^ of patients received low tidal volumes, with one study finding a median tidal volume of 8.8 mL/kg of ideal body weight.^34^ Similar to our findings, a national survey of EDs from 2014 found that 73% of respondents reported that patients routinely received mechanical ventilation for several hours in the ED.^35^ Emergency physicians noted a lack of literature to guide mechanical ventilation specifically in the ED, but 100% of respondents were willing to adopt an intervention that could decrease the incidence of ARDS,^35^ such as low tidal volume ventilation. These prior findings demonstrate opportunities for improvement in mechanical ventilation in the ED, as well as a willingness to adopt such interventions on the part of emergency physicians.

Throughout this current study, several parallels emerged between attendings and the previous study of EM residents. In addition to having the same score on the assessment instrument, a similar proportion of residents reported few hours of education with a high frequency of caring for ventilated patients. Also, 78% of residents felt that ventilator management was the responsibility of the RT. For the residents, level of comfort correlated with test score, much like the attendings. As with their attendings, the residents’ comfort increased with available hours of education on ventilation.

These findings support the importance of education in residency, as increased hours of education improve the assessment score and increase comfort. Perhaps more importantly, education in residency appears to have lasting effects, as emphasis on mechanical ventilation in residency correlated with an improved attending performance on the assessment tool. Additionally, in our prior study, we found that residents had sufficient knowledge, but were not actively applying that knowledge by ceding opportunities to manage the ventilator to RTs. We hypothesized that there is an opportunity to improve emergency physicians’ familiarity and comfort with ventilators by encouraging more active involvement with ventilator management decisions in the ED. While emergency physicians performed well on the assessment instrument, there is potential benefit of active ventilator management and bedside education for both EM residents and attendings in improving knowledge and comfort with ventilated patients. This concept is supported by this study, as active management of the ventilator is associated with confidence in caring for these patients.

## LIMITATIONS

Although our results are similar to a prior study of EM residents, our response rate in the current study was lower at 54%, with 211 respondents. Therefore, our results may have been influenced by non-responder bias and may be underpowered to detect significant differences. Physicians’ interest in the topic of mechanical ventilation may have influenced participation in this study, with interested physicians being more likely to complete the knowledge assessment tool and less-interested attendings being less likely to participate and complete the tool. A study of patient outcomes related to management of mechanical ventilation was beyond the scope of this limited study, and therefore, the impact on patients is not known. Finally, our multicenter study involved academic emergency physicians in the northeastern U.S., and the generalizability of our results to other settings, regions or countries is not known. This current study did not test the effects of an educational intervention on knowledge of mechanical ventilation. Assessing the value of education on ventilation for emergency physicians is an important future direction for study.

## CONCLUSION

In this sample, we noted that attending physicians report caring for mechanically ventilated patients in the ED quite frequently; however, they also reporting having few educational opportunities regarding mechanical ventilation, with 75% stating that they received three hours or fewer over the last year. The majority of respondents identified a respiratory therapist as being primarily responsible for ventilator management, with few describing this role as belonging to an emergency physician.

Performance on our mechanical ventilation knowledge assessment was moderate, with an average score of 73.4%. A higher score on the assessment portion correlated with prior emphasis on mechanical ventilation in the physician’s own residency, completion of an EM residency, and self-reported comfort in caring for ventilated patients. Physicians’ comfort was associated with the frequency of ventilator changes and EM management of ventilation, as well as hours of recent education. These findings suggest that education in residency may have lasting effects on future performance, and active participation in decisions regarding mechanical ventilation management, as well as educational opportunities, can increase confidence in caring for these critically ill ED patients.

## Figures and Tables

**Table 1:** Emergency medicine (EM) attendings’ self-reported education and experience regarding mechanical ventilation.

Survey question	Respondents (%)
How long have you been an EM attending?	
0–2 years	36 (17.1)
3–5 years	33 (15.6)
5–10 years	56 (26.5)
10–15 years	37 (17.5)
>15 years	49 (23.2)
Have you completed an EM residency?	
Yes	189 (89.6)
No	22 (10.4)
Have you trained in another residency besides EM?	
No	171 (81.4)
Internal medicine	16 (7.6)
Surgery	8 (3.8)
Other	15 (7.1)
Did you complete a fellowship after EM residency?*	
No	111 (52.6)
Ultrasound	20 (9.5)
Toxicology	3 (1.4)
Pediatrics	10 (4.7)
Emergency medical services	9 (4.3)
Critical care	10 (4.7)
Wilderness medicine	2 (1.0)
Research	15 (7.1)
Other	32 (15.2)
Was mechanical ventilation an emphasized topic during your EM residency training?	
Never emphasized	8 (3.8)
Rarely emphasized	71 (33.6)
Sometimes emphasized	85 (40.3)
Often emphasized	21 (10.0)
Frequently emphasized	8 (3.8)
Not applicable - I did not do an EM residency	18 (8.5)
How many hours of instruction have you received on mechanical ventilation from other EM sources (EM articles, discussion in EM journal clubs, EM lectures/conferences, etc) in the last year?	
0–1	98 (46.4)
2–3	60 (28.4)
4–5	13 (6.2)
More than 5	33 (15.6)
Don’t know	7 (3.3)
How often do you care for mechanically ventilated patients in the emergency department?	
Never	0 (0.0)
Rarely (1–3 patients/month)	72 (34.1)
Often (4–9 patients/month)	97 (46.0)
Frequently (>10 patients/month)	39 (18.5)
Don’t know	3 (1.4)
How often do mechanically ventilated patients in the emergency department (ED) have adjustments made to the ventilator while still in the ED?	
Never	0 (0)
Rarely (1–3 patients/month)	118 (55.9)
Often (4–9 patients/month)	68 (32.2)
Frequently (>10 patients/month)	14 (6.6)
Don’t know	11 (5.2)
How often do you feel comfortable managing mechanical ventilation and troubleshooting issues with ventilated patients in the ED?	
Never	3 (1.4)
Rarely	78 (37.0)
Often	96 (45.5)
Frequently	30 (14.2)
Don’t know	4 (1.9)
Who primarily directs changes to the mechanical ventilator for intubated patients in your ED?	
Respiratory therapist	145 (68.7)
Nurse	0 (0)
EM resident	15 (7.1)
EM attending	44 (20.9)
Physician not affiliated with the ED (ICU, pulmonologist, etc)	4 (1.9)
Don’t know	3 (1.4)

* Some respondents completed more than 1 fellowship.

*ICU,* intensive care unit

**Table 2:** Correlations between survey responses and total score on assessment tool.

Characteristics of training program and experience	Correlation with total test score (ρ)	P-value
Years as emergency medicine (EM) attending	−0.110	0.114
Training in another residency	−0.111	0.109
Fellowship training	0.023	0.743
Emphasis on mechanical ventilation in EM residency	0.260	<0.001*
Hours of mechanical ventilation education	0.093	0.182
Frequency of managing ventilated patients	0.105	0.129
Frequency of ventilator changes in the emergency department	0.055	0.431
Level of comfort with managing mechanically ventilated patients	0.356	<0.001*
EM management of mechanical ventilator	0.133	0.053
Completion EM residency	0.212	0.002*
Program affiliation	0.152	0.027*

* Statistically significant correlation (2-tailed).

**Table 3:** Correlations between candidate predictor variables and attending physician comfort with managing mechanically ventilated patients.

Characteristics of training program and experience	Correlation with comfort (ρ)	P-value
Years as emergency medicine (EM) attending	−0.070	0.312
Training in another residency	0.092	0.185
Fellowship training	−0.066	0.342
Emphasis on mechanical ventilation in EM residency	0.261	<0.001*
Hours of mechanical ventilation education	0.250	<0.001*
Frequency of managing ventilated patients	0.134	0.052
Frequency of ventilator changes in the emergency department	0.147	0.033*
EM management of mechanical ventilator	0.258	<0.001*
Completion of EM residency	0.088	0.202
Program affiliation	0.001	0.985

* Statistically significant correlation (2-tailed).
